# Metal Films on Two-Dimensional Materials: van der Waals Contacts and Raman Enhancement

**DOI:** 10.1021/acsami.3c15598

**Published:** 2024-02-06

**Authors:** Maheera Abdul Ghani, Soumya Sarkar, Jung-In Lee, Yiru Zhu, Han Yan, Yan Wang, Manish Chhowalla

**Affiliations:** Department of Materials Science & Metallurgy, University of Cambridge, 27 Charles Babbage Road, Cambridge CB3 0FS, U.K.

**Keywords:** van der Waals contacts, indium metal, transition-metal dichalcogenides, graphene, localized surface plasmon resonance

## Abstract

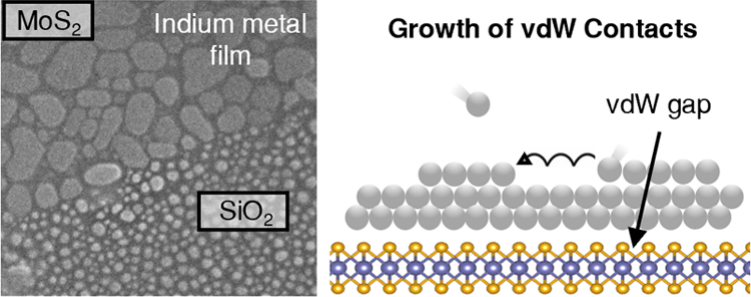

Electronic devices based on two-dimensional (2D) materials will need ultraclean and defect-free van der Waals (vdW) contacts with three-dimensional (3D) metals. It is therefore important to understand how vdW metal films deposit on 2D surfaces. Here, we study the growth and nucleation of vdW metal films of indium (In) and non-vdW metal films of gold (Au), deposited on 2D MoS_2_ and graphene. In follows a 2D growth mode in contrast to Au that follows a 3D growth mode. Atomic force microscopy (AFM) and scanning electron microscopy (SEM) were used to image the morphology of metal clusters during growth and quantify the nucleation density. As compared to Au, In atoms exhibit nearly 50 times higher diffusivity (3.65 × 10^–6^ μm^–2^ s^–1^) and half the nucleation density (64.9 ± 2.46 μm^–2^), leading to larger grain sizes (∼60 nm for 5 nm In on monolayer MoS_2_). The grain size of In can be further increased by reducing the 2D surface roughness, while the grain size for Au is limited by its high nucleation density due to the creation of interface defects during deposition. The vdW gap between In and MoS_2_ and graphene leads to strong enhancement (>10^3^) in their Raman signal intensity due to localized surface plasmon resonance. In the absence of a vdW gap, the plasmon-mediated enhancement in Raman does not occur.

## Introduction

Achieving clean electrical contacts between two-dimensional (2D) semiconductors and metal electrodes is crucial for enabling high-performance electronic devices.^1−4^ A promising approach is to use van der Waals (vdW) contacts that are characterized by a 2–4 Å vdW gap between the metal and the 2D surface.^5^ vdW contacts based on transferred graphene,^6^ transferred metal films,^7^and direct deposition using indium (In) have been demonstrated.^8^ Direct deposition of vdW metal contacts is scalable and is technologically relevant. However, an understanding of the morphology and growth of vdW metals on 2D surfaces is currently lacking and could provide insights into developing good electrical contacts.

In this work, we study how In and gold (Au) metal films grow on 2D MoS_2_ and graphene. When deposited using standard laboratory evaporation techniques, we have demonstrated^8^ that In forms vdW contacts on MoS_2_ while Au leads to damage at the interface with MoS_2_.^9^ We observe that defects on the surface during Au deposition lead to higher nucleation density of Au islands with small grain sizes. In contrast, the low nucleation density of In due to fewer defects introduced during metal deposition leads to large continuous grains. The nucleation density of In grains depends on the roughness of the substrate (in this case, SiO_2_) underneath the 2D material. The metal film nucleation and growth are correlated to the lower roughness of MoS_2_ on SiO_2_ compared to graphene on SiO_2_ and SiO_2_ alone. We demonstrate that increasing the number of MoS_2_ layers reduces the influence of the SiO_2_ substrate roughness so that the diffusivity and diffusion length of In atoms increase and activation energy for the diffusion of In atom decreases—as reflected in the 5-fold decrease of nucleation density (∼12 μm^–2^) and an increase in grain size (∼180 nm) for the bulk MoS_2_ substrate. In contrast, the diffusivity, diffusion length, and activation energy for the diffusion of Au on MoS_2_ remain largely unchanged because the deposition of Au on MoS_2_ leads to damage, which has the effect of increasing the roughness. Finally, we demonstrate that the vdW-contacted thin In films (5 nm) with a grain size of ∼60 nm on MoS_2_ leads to significant enhancement (enhancement factor (EF) > 10^3^) in the intensity of Raman modes of 2D materials due to localized surface plasmon resonance. Such enhancement effects are absent in the case of non-vdW Au metal thin films.

## Results and Discussion

**Figure 1:**
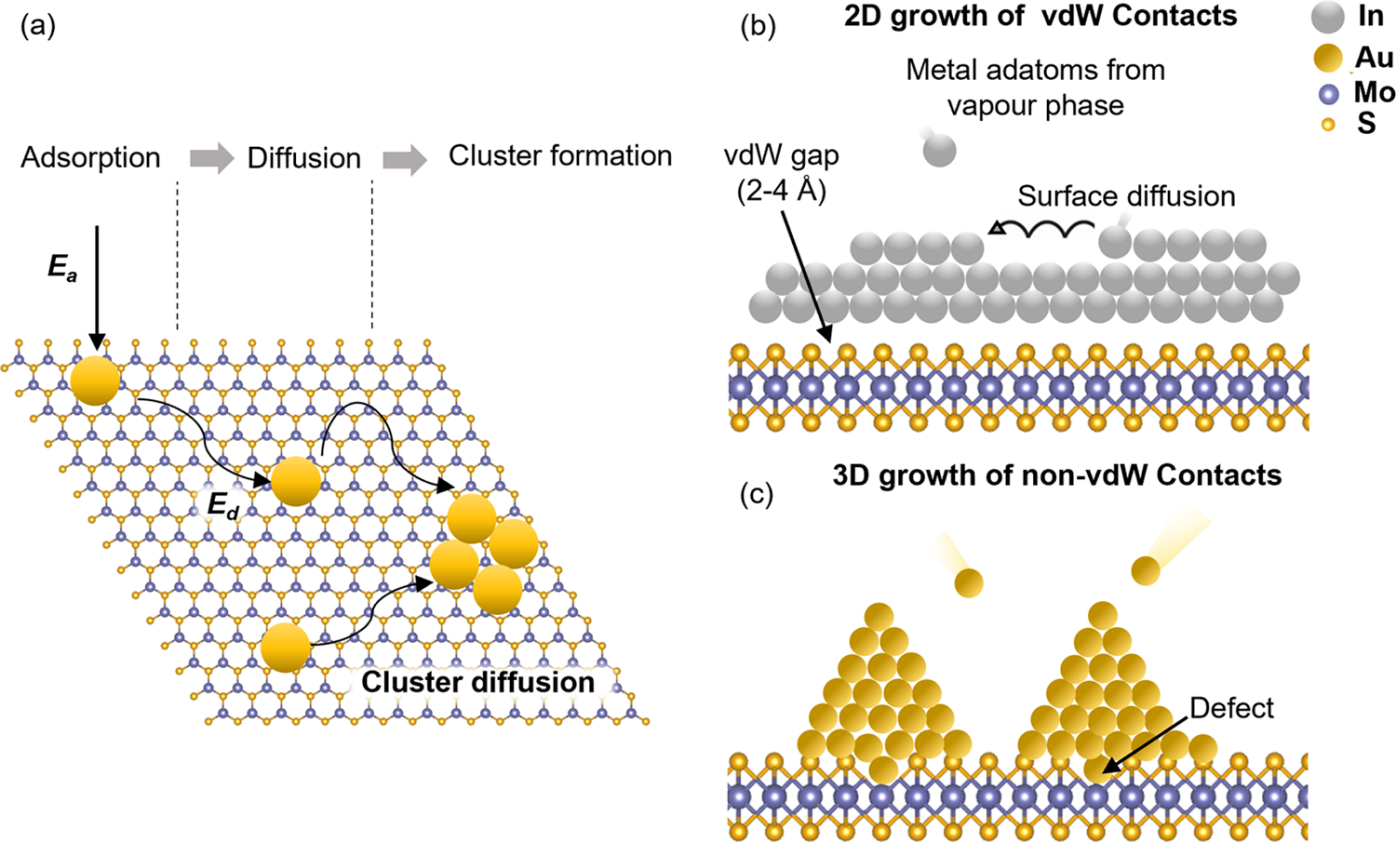
Schematic describing (a) the growth of metal thin film on 2D MoS_2_ that involves adsorption of the evaporated metal, adatom and cluster diffusion, and nucleation. (b) 2D growth of In-based vdW and (c) 3D growth of Au-based non-vdW contacts on MoS_2_.

Metal contacts are generally deposited using thermal or electron beam evaporation.^10^ As the evaporated metal atoms deposit on the substrate, several important processes (as shown in the schematic in Figure 1a) govern the nucleation and growth of metal films.^11−13^

A metal atom from the vapor phase is first adsorbed onto the substrate with atomic adsorption energy (*E*_a_).^14^ The adsorbed atom or adatom then diffuses on the surface with diffusion energy (*E*_d_) until it encounters a low-energy nucleation site. During diffusion on the substrate surface, multiple adatoms can combine to form a cluster before reaching a nucleation site.^15^ Nucleation begins when the cluster of atoms reaches a critical radius. Beyond this, the cluster is stable and grows laterally to form grains that eventually coalesce with their neighbors to form a continuous film.^12^

The surface diffusion of adatoms is influenced by the degree of interaction with the substrate.^16,17^ 2D materials are chemically inert and relatively smooth, with few available sites for nucleation. Therefore, the interaction of 2D materials with metal adatoms is weak,^18,19^ which implies high diffusivity and long diffusion lengths leading to the formation of large clusters before encountering a nucleation site on the surface. As the nucleation site density is low, larger grains grow to achieve a continuous film. The type of metal also plays a role in nucleation and growth of the films on 2D materials.^20^ Soft metals such as In form vdW contacts with little or no chemical interaction with the 2D material. Previously, we have demonstrated atomically sharp and defect-free vdW interface using cross-sectional scanning transmission electron microscopy (STEM) between In metal contacts and MoS_2_.^8^ Such vdW interactions lead to low *E*_*a*_ on the surface with a longer length for surface diffusion and low surface diffusion barrier. As a result, In adatoms tend to form large clusters before settling at nucleation sites. In contrast, the evaporated atoms of relatively refractory metals such as Au possess high kinetic energy that introduces defects in atomically thin 2D materials during deposition.^7^ These defects reduce the surface diffusion lengths and act as low-energy nucleation sites for grain growth. Consequently, a higher defect density in the 2D material will lead to higher metal nucleation density and smaller grain size.^21^ Thus, in the case of Au deposition on MoS_2_, very small grains are observed because of the numerous defects created by Au atoms during deposition. The proximity of the nucleation sites means that smaller grains are required for their coalescence into a continuous film. Overall, the interaction strength between the metal adatom and the substrate (*E*_a_) and the metal–metal bonding strength or cohesive energy (*E*_c_) of the bulk metal are factors that determine metal thin film growth modes (Figure 1b,c). The dimensionality of the metal film growth (i.e., 2D or 3D growth) is governed by the *E*_a_/*E*_c_ ratio.^22^ Larger *E*_a_/*E*_c_ follows 2D growth morphology and forms a uniform film as shown in Figure 1b, while a smaller ratio can lead to 3D island-like growth. The ratio of the adsorption energy to the cohesive energy (*E*_a_/ *E*_c_) for In/graphene (0.322 eV)^23^ is about 12 times higher than for Au/graphene (0.025 eV)^23−25^ and is 0.45 eV for In/MoS_2_^26^ and 0.17 eV for Au/MoS_2_^26−28^—resulting in 2D-like growth for In and 3D island growth for Au.

**Figure 2:**
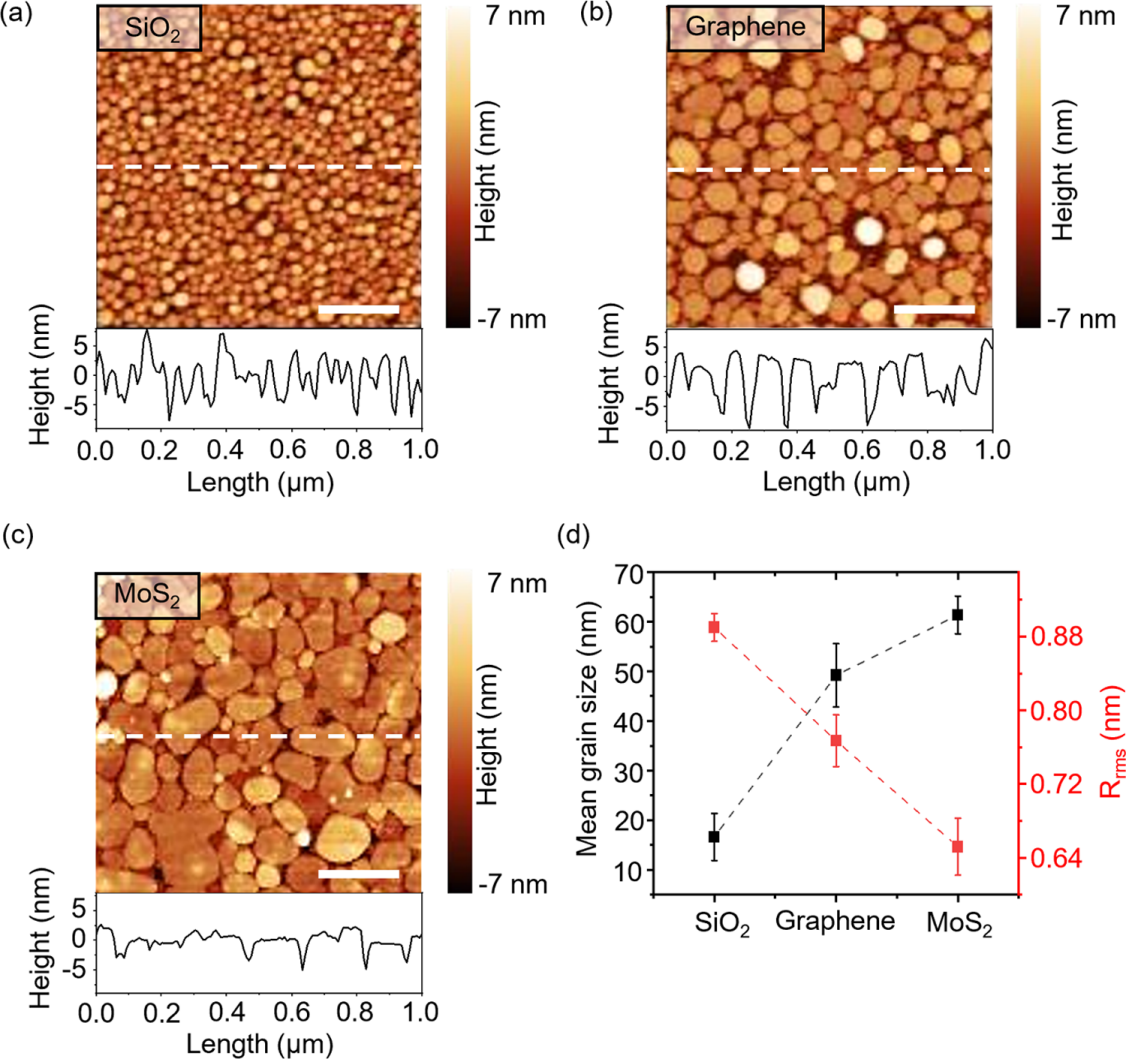
Substrate-dependent morphology of In metal deposition. AFM (1 × 1 μm^2^) images and height profiles of a 5 nm thick In metal film deposited on different substrates. (a) SiO_2_/Si substrate, (b) monolayer graphene, and (c) monolayer MoS_2_ on the SiO_2_/Si substrate. The scale bar is 250 nm. (d) Mean grain size and *R*_rms_ (before metal deposition) plotted against three different substrates. Error bars in panel (d) represent the standard deviation across 5 AFM images (1 × 1 μm^2^).

Monolayer and few-layer flakes of MoS_2_ and graphene were mechanically exfoliated from a bulk crystal on the SiO_2_/Si substrates. Thin films of In and Au of thicknesses ranging from 2 to 50 nm were deposited using electron beam evaporation. Atomic force microscopy (AFM) images from 5 nm thick In films deposited on SiO_2_, graphene, and MoS_2_ are shown in Figure 2a–c along with their corresponding height profiles (along the white dashed lines in the AFM images). The average grain size of In on different substrates has been correlated with the substrate roughness in Figure 2d. Bare SiO_2_ roughness was measured to be ∼0.89 nm, with an average In grain size of 17 nm. The average grain size of In on graphene is around 50 nm with a roughness of ∼0.76 nm. For MoS_2_, the average grain size (average grain diameter) of In is 60 nm, and the surface roughness is ∼0.65 nm. (see Section 1 in the Supporting Information (SI) for AFM images of the samples before metal deposition). The root-mean-square roughness (*R*_rms_) value calculated for SiO_2_ is consistent with previous studies.^29^ The relatively high roughness of SiO_2_ suppresses adatom surface diffusion and provides ample opportunities for nucleation and growth of grains.^16,17^

Related parameters such as nucleation density (*N*, represented as grains per μm^2^), diffusivity, diffusion length, and activation energy for diffusion can be estimated from mean field diffusion theory.^17,30,31^ For 2D island growth, *N* is proportional to diffusivity (*D*) of the metal atom as , where *i* is the critical radii for nucleation.^20,30,32^ The diffusivity is related to the activation energy of diffusion (*E*_d_), and we can estimate using , where *k* is Boltzmann’s constant, *T* is temperature, and Δ*E* is the diffusion barrier.^17^ Combining these proportionalities allows nucleation density to be related to the activation energy via . The diffusion length of adatom on the surface is .^17^ We estimate *N* from the number of individual grains per unit area in the AFM images. These parameters and the *R*_rms_ values for In on MoS_2_, graphene, and SiO_2_ are summarized in Table 1 (Supporting Section 2 summarizes the results of our calculations of diffusivity and related parameters for both *i* = 1 and 2).

**Table 1:** Calculated Values of Diffusivity (*D*), Diffusion Length (*L*_D_), and Activation Energy for Diffusion (*E*_d_) for Three Different Substrates (*i* = 1)

**substrate**	**nucleation density (*****N*****) (μm**^**–2**^**)**	**diffusivity (*****D*****)(μm**^**–2**^ **s**^**–1**^**)**	**diffusion length (*****L***_**D**_**) (μm)**	**activation energy for diffusion, (*****E***_**d**_**) (eV)**
1L MoS_2_	64.9 ± 2.46	3.65 × 10^–6^	0.124	0.313
1L graphene	104.7 ± 3.21	8.71 × 10^–7^	0.098	0.349
SiO_2_	426.7 ± 2.83	1.28 × 10^–8^	0.048	0.454

**Figure 3:**
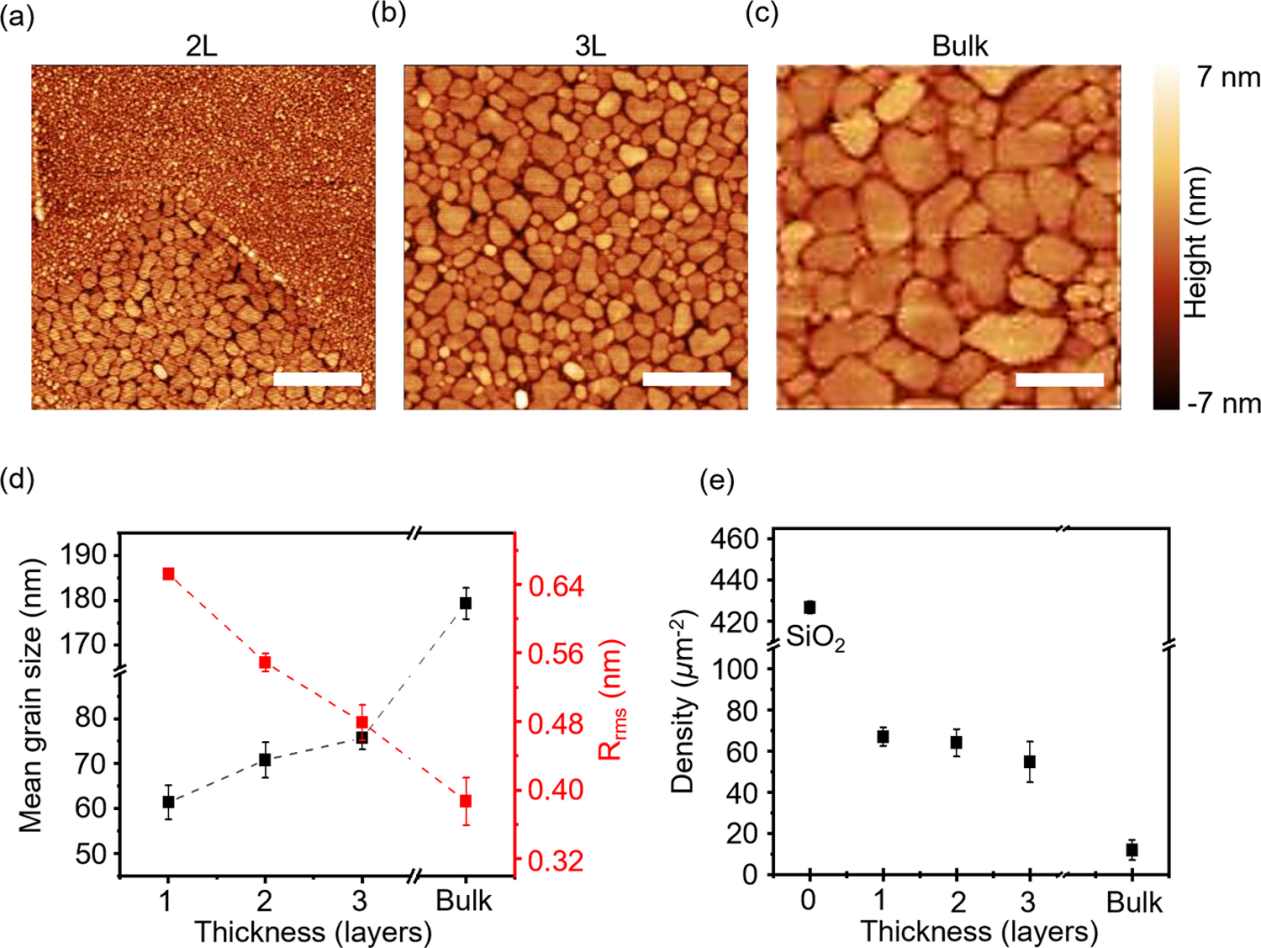
Growth of In metal thin films on different thicknesses of MoS_2_. AFM (2 × 2 μm^2^) images of 5 nm In deposited on (a) 2L, (b) 3L, and (c) bulk MoS_2_. Scale bar = 500 nm. (d) Mean grain size and *R*_rms_ (before metal deposition) of MoS_2_ flakes plotted against sample thickness. (e) In nucleation density on MoS_2_ plotted against sample thickness. Error bars in parts (d) and (e) represent the standard deviation across 5 AFM images (2 × 2 μm^2^).

Coating the SiO_2_ with graphene reduces the roughness but the atomically thin nature of graphene replicates some of the topology of SiO_2_.^33^ MoS_2_ (thickness = 0.61 nm) is twice as thick as graphene (thickness = 0.33 nm) and largely masks the roughness of the underlying SiO_2_. To investigate the effect of increasing thickness of the 2D material on the metal grain size, 5 nm In and Au were deposited on one layer (IL), two layer (2L), three layer (3L), and >50 layers of MoS_2_. The AFM images in Figure 3a–c show the evolution of In grains as the thickness of MoS_2_ is increased from 2L to bulk. The increase in grain size as a function of the MoS_2_ layer thickness (from 60 nm in 1L to 180 nm in bulk) has been correlated with the decrease in its surface roughness (from 0.65 nm in 1L to 0.38 nm in bulk, as shown in Figure S1 of the SI) in Figure 3d. Notably, the grain size of In on bulk MoS_2_ is significantly larger, which is consistent with the 5 times lower nucleation density (see Figure 3e), 3 times longer diffusion lengths, nearly 2 orders of magnitude higher diffusivity than 1L MoS_2_, and lowest activation energy for diffusion listed in Table 2. This suggests that MoS_2_ that is much thicker than 3 layers can suppress the effects of the SiO_2_ substrate. The corresponding trends of the In grain size as a function of graphene thickness are shown in Section 3 of the SI.

**Table 2:** Calculated Values for Diffusivity (*D*), Diffusion Length (*L*_D_), and Activation Energy for Diffusion (*E*_d_) for Four Different Thicknesses of MoS_2_ (*i* = 1)

**substrate**	**nucleation density (*****N*****) (μm**^**–2**^**)**	**diffusivity (*****D*****) (μm**^**–2**^**s**^**–1**^**)**	**diffusion length (*****L***_**D**_**) (μm)**	**activation energy for diffusion, (*****E***_**d**_**) (eV)**
1L MoS_2_	64.9 ± 2.46	3.65 × 10^–6^	0.124	0.313
2L MoS_2_	61.0 ± 3.62	4.41 × 10^–6^	0.128	0.308
3L MoS_2_	52.7 ± 9.84	6.83 × 10^–6^	0.138	0.297
bulk MoS_2_	12.5 ± 7.86	5.12 × 10^–4^	0.283	0.189

**Figure 4:**
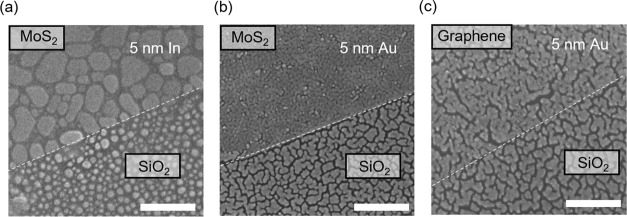
SEM images of surface morphologies of (a) a 5 nm In film and (b) a 5 nm Au film deposited on monolayer MoS_2_. (c) 5 nm Au film deposited on monolayer graphene. Scale bar is 200 nm.

The SEM images of 5 nm In film on MoS_2_ (and SiO_2_) in Figure 4a further confirm that In forms large grains on MoS_2_ as was observed by AFM. In contrast, the deposition of Au on MoS_2_ demonstrates a completely different morphology as shown in Figure 4b. The Au thin film on MoS_2_ shows poorly defined, interconnected grains. The morphology of the Au film on SiO_2_ shows randomly shaped grains. The number of grains per unit is significantly larger (109 μm^–2^) for Au on MoS_2_—suggesting higher nucleation sites. This is consistent with the fact that the deposition of Au on MoS_2_ leads to the creation of significant defects on the surface. The presence of defects leads to strong adsorption of adatoms on the surface, resulting in a high density of nucleation sites. The high nucleation density coupled with the high *E*_c_ of Au atoms leads to a relatively dense island-like growth of Au films on MoS_2_. The morphology of Au on graphene is shown in Figure 4c, where regions of dense growth can be seen along with regions where a Au film is absent. The dendrite-like nonuniform growth of Au on graphene suggests heterogeneous nucleation. Graphene is more tolerant of metal deposition,^34^ so defects are less likely to be produced during Au film growth compared to MoS_2_ leading to less nucleation sites and patchy growth as observed. This is consistent with the relatively high diffusivity 3.65 × 10^–6^ μm^–2^ s^–1^ of In atoms on MoS_2_ compared to 7.40 × 10^–8^ μm^–2^ s^–1^ for Au atoms. We observe 2D growth even for thicker In films. As shown in Figure S4 of the SI, as the thickness of the In film on MoS_2_ is increased from 5 to 15 nm, the grain size increases from 61 to 135 nm due to a lateral coalescence of adjacent grains.

To further understand the role of defects in determining the growth morphology of metal films on 2D surfaces, we deposited In and Au on thermally annealed MoS_2_ with additional defects.^35^ For In, the additional defects increase the nucleation density by 50%. However, the nucleation density for Au on defective MoS_2_ is similar to what was observed on pristine MoS_2_, as the nucleation of Au is dominated by defects created on the MoS_2_ surface during the deposition process (see Section 5 in the SI).

**Figure 5:**
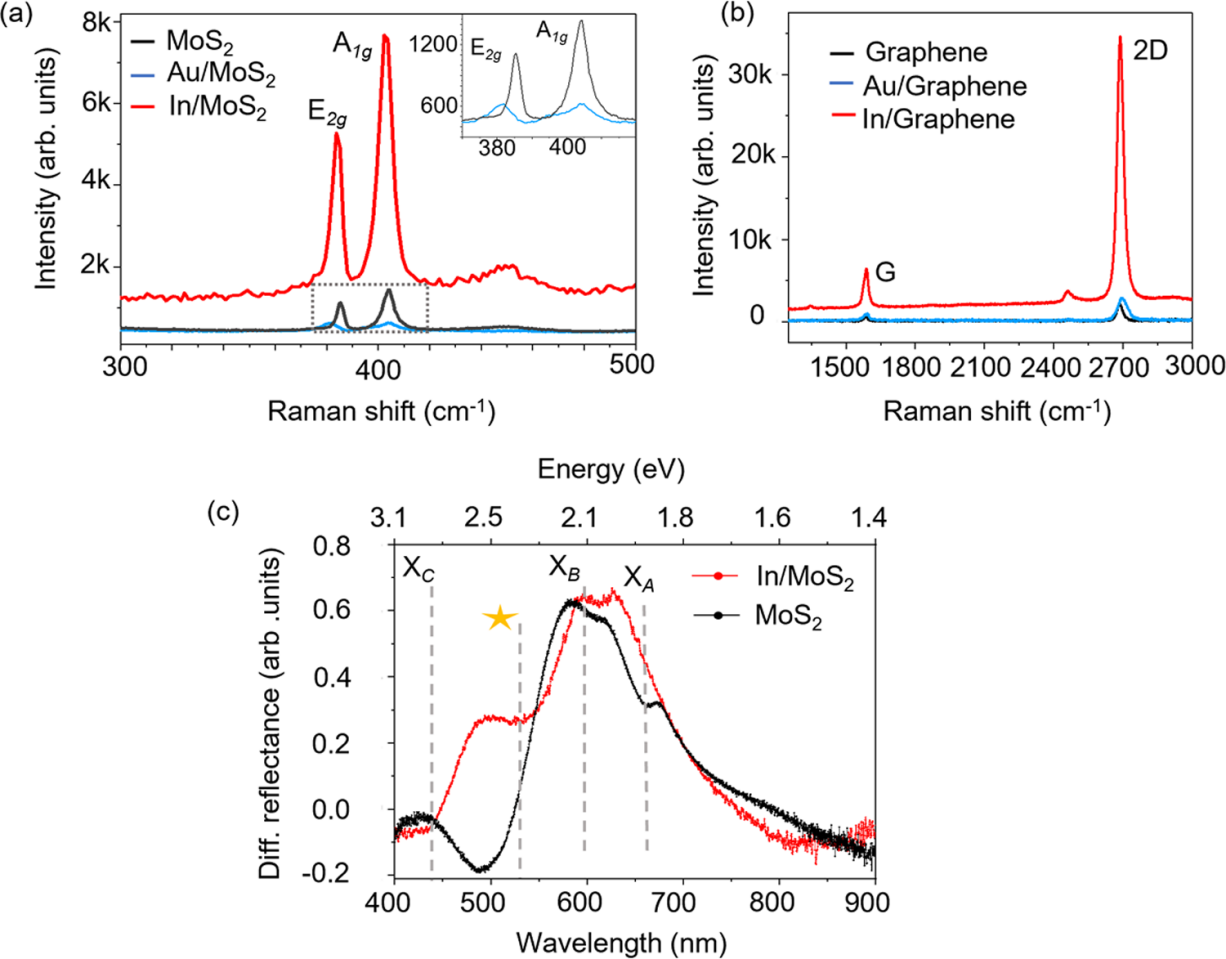
Raman spectra of pristine (a) 1L MoS_2_ and (b) 1L graphene compared to the Raman spectra collected after deposition of 5 nm Au and 5 nm In thin films. (c) Differential reflectance of 1L MoS_2_/SiO_2_ collected before and after 5 nm In deposition.

Raman spectroscopy can be used as a tool to investigate strain, defects, and doping in 2D materials induced during the metal deposition process.^36^ Raman spectra of monolayer graphene and MoS_2_ as well as graphene and MoS_2_ with 5 nm thick In and Au films are shown in Figure 5a,b. Raman spectra of pristine 1L MoS_2_ show characteristic E_2g_ and A_1g_ phonon modes. After 5 nm of Au deposition, Raman peak intensities of A_1g_ and E_2g_ phonon modes in MoS_2_ display a decrease in the intensity of 60 and 40%, respectively, and full width at half-maximum (fwhm) values double from 2.9 to 6.4 cm^–1^ for E_2g_ and from 5.02 to 10.25 cm^–1^ for A_1g_. We observe softening of Raman modes in the case of Au on MoS_2_. Specifically, we observe softening of the E_2g_ Raman mode of MoS_2_ by 4 cm^–1^ after Au metal deposition due to strain.^37^ Raman spectra collected from 5 nm In-coated MoS_2_ show an order of magnitude increase in the intensity of E_2g_ and A_1g_ phonon modes. Similar enhancement in the intensities of G and 2D phonon modes of graphene when coated with 5 nm In is also observed (Figure 5b). Such an enhancement is absent when a 5 nm Au film is deposited on graphene. Graphene has a more stable lattice and is relatively more chemically inert.^38^ As a result, we do not observe significant differences in the Raman spectra of graphene before and after Au deposition. The intensity ratio of the 2D peak to G peak (*I*_2D_/*I*_G_) changes from 3.2 to 2.9 after Au deposition and they blue shift by 6 and 2 cm^–1^, respectively, which indicates change in the carrier concentration from 10^12^ to 10^13^ cm^–2^.^39^ Such shifts of the phonon modes are negligible for In-based vdW metal contact on 2D materials.

The observed enhancement in Raman after a 5 nm In deposition could be due to localization of surface plasmons in In grains that electrostatically couple across the vdW gap with phonons in graphene and MoS_2_. The absence of defects at the metal–semiconductor interface and the absence of charge transfer due to the vdW gap ensures good plasmon–phonon coupling. The enhancement factor of the Raman modes is estimated to be >10^3^ for MoS_2_ and graphene (see Section 6 in the SI).^40^ The differential reflectance spectra from monolayer MoS_2_ on the SiO_2_/Si substrate before and after a 5 nm In deposition are shown in Figure 5c. The characteristic A and B excitonic features of MoS_2_ 1.85 and 2.05 eV, respectively, are observed (dashed lines). A new reflectance dip is observed near 2.5 eV (500 nm) (starred) after a 5 nm In deposition on MoS_2_ which indicates absorption by localized plasmons on In metal grains that are responsible for the observed enhancement in Raman. The deposition of Au does not introduce any new resonance features (Figure S8 in the SI). The observed Raman enhancement is maximum for 5 nm thick In metal films and reduces for increasing metal thickness, which could be due to delocalization of plasmons along the coalesced metal grains.

## Conclusions

We demonstrate that In metal film deposition on MoS_2_ and graphene follows a 2D growth mechanism to form large grains. In contrast, the deposition of Au introduces defects and disorder in 2D materials during growth, leading to high nucleation density and 3D island growth. The weak interaction with the 2D materials along with a decrease in surface roughness allows large grains of In to form. The presence of a vdW gap between In and 2D materials prevents charge transfer from occurring. This allows localized plasmons excited in the In grains to couple with phonons in MoS_2_ and graphene to enhance their Raman signal intensity by >10^3^. This is one of the first demonstrations of using the vdW gap between metal particles and 2D materials to increase the coupling between plasmons and phonons.

## Experimental Section

### MoS_2_ and Graphene Sample Preparation

MoS_2_ and graphene samples were obtained by mechanical exfoliation^41^ from bulk crystals (2D Semiconductors). The flakes were exfoliated on a 285 nm thick SiO_2_ on a Si substrate using adhesive blue tape. Prior to this, the substrates were thoroughly cleaned for 15 min in acetone, isopropyl alcohol (IPA), and deionized water to remove any organic residues. The thicknesses and surface topography of flakes were confirmed by optical contrast, AFM, and Raman spectroscopy.

### In and Au Metal Deposition

In films were deposited using a standard electron beam evaporator system at a base pressure of <2 × 10^–7^ Torr. Before each deposition, the metal was heated for 5 min to remove any surface oxides or contaminations. The distance between the crucible containing metal and substrate was kept at 70 cm to minimize the kinetic energy of the evaporated metal atoms on the sample. The sample holder remained at or close to room temperature during deposition. Au metal films were prepared without the precautions mentioned above to deliberately create non-van der Waals contacts through physical damage.

### AFM and SEM

AFM imaging was performed using a Bruker Icon system in ambient conditions at a scan rate of 0.6 Hz. The AFM images were analyzed using the Gwyddion 2.60 software package to determine grain size (using the Watershed thresholding algorithm) and surface roughness. For SEM measurements, the samples were imaged in an FEI Nova NanoSEM operated at 10 kV. The samples were grounded to avoid charging-induced damage.

### Raman Spectroscopy

Raman spectra were acquired using a Renishaw InVia Raman microscope with a 514 nm laser excitation and a 2400 l/mm grating. The laser power was kept below 100 mW to avoid damage to the flakes.

## Supporting Information Available

Section 1: atomic force microscopy (AFM) images of MoS_2_ flakes before metal deposition; Section 2: estimation of diffusivity, diffusion length, and activation energy for diffusion; Section 3: In metal deposition on different thicknesses of graphene; Section 4: effect of thicker In metal deposition on grain morphology and Raman spectra; Section 5: growth morphology of In and Au metal films on defective MoS_2_; Section 6: Raman enhancement factor (EF) calculation; and Section 7: differential reflectance of 1L MoS_2_/5 nm Au (PDF)
