# Supplementary material for: Metal Films on Two-Dimensional Materials: van der Waals Contacts and Raman Enhancement
Source: ACS Appl Mater Interfaces. 2024 Feb 6;16(6):7399–405. doi: 10.1021/acsami.3c15598 (PMC10875649; doi:10.1021/acsami.3c15598)
Supplement: Supplementary file 1 — am3c15598_si_001.pdf [file am3c15598_si_001.pdf]

Supporting Information

Metal Films on Two-Dimensional Materials: van der Waals Contacts and Raman Enhancement

Maheera Abdul Ghani[1], Soumya Sarkar[1], Jung-In Lee[1], Yiru Zhu[1], Han Yan[1], Yan Wang[1], Manish Chhowalla[1*]*

[1]Department of Materials Science & Metallurgy, University of Cambridge, 27 Charles Babbage Road, Cambridge CB3 0FS, UK.

*Correspondence should be addressed to ss2806@cam.ac.uk, mc209@cam.ac.uk

Section 1: Atomic Force Microscopy (AFM) images of MoS_2 flakes before metal deposition

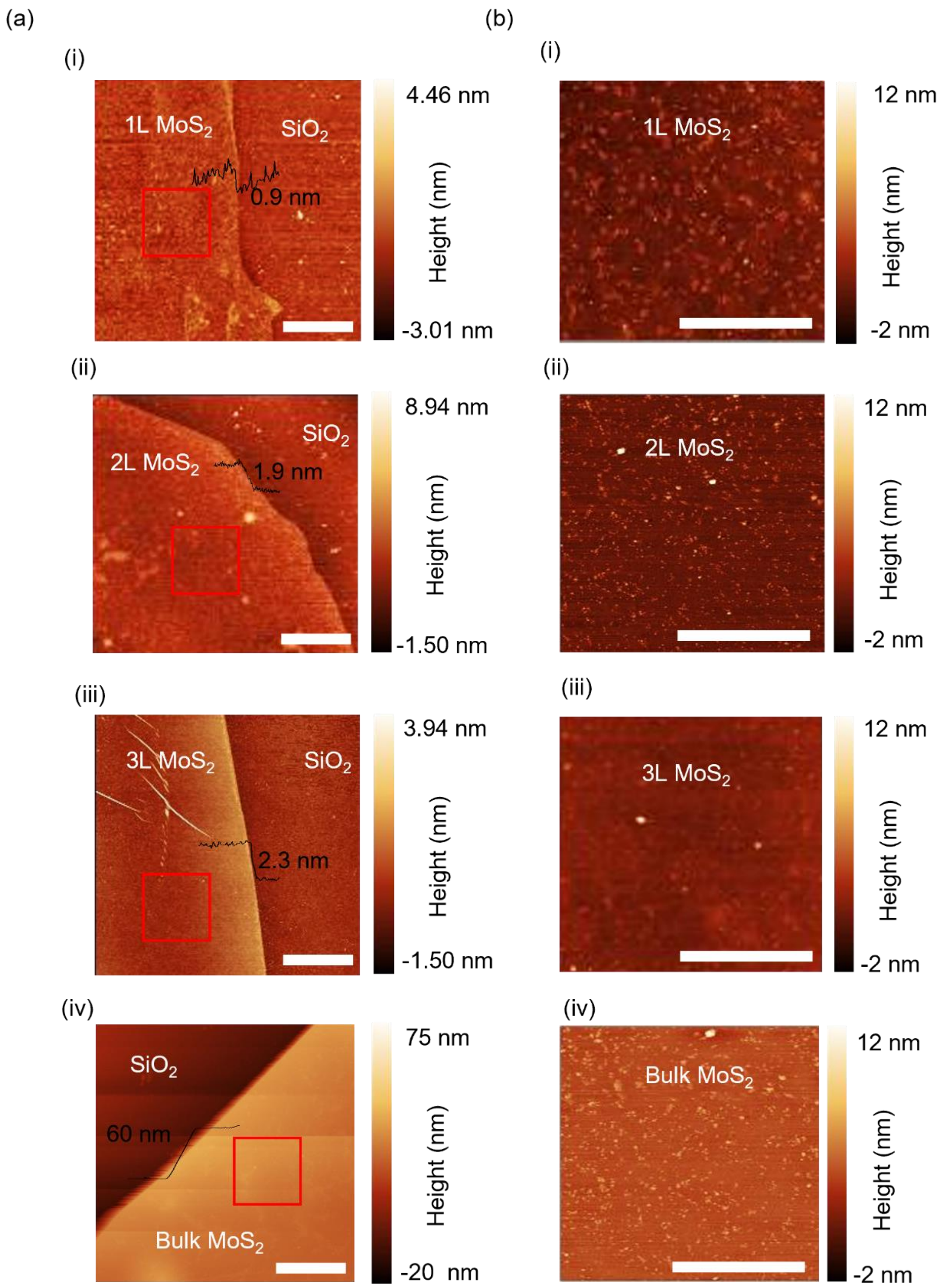

Figure S1. (a) AFM images (2×2 μm^2) of (i) 1L, (ii) 2L, (iii) 3L, (iv) Bulk MoS_2 substrates before metal deposition. Scale bar is 500 nm. AFM line profiles overlayed on the images indicate the thickness of the flake (b) AFM images (500×500 nm^2) of bare MoS_2 substrates regions marked inside red box from (a). Scale bar is 250 nm.

Section 2: Estimation of diffusivity, diffusion length and activation energy for diffusion

We estimated the values of diffusivity, diffusion length, and activation energy using mean field diffusion and thermodynamic nucleation theories.[1] We estimate nucleation density (N, grains per μm^2), from the number of individual grains per unit area using AFM images (Gwyyidion 2.60 software). The diffusivity (D) of In considering 2D growth is related to nucleation density as:

$$N = \left(\frac{1}{D}\right)^{\frac{i}{i+2}} \tag{1}$$

Where, i is the cluster size[1]. For i = 1, and N= 64.9 ± 2.46 μm^{-2} (for 1L MoS_2), diffusivity (D) was calculated as:

$$64.9 = \left(\frac{1}{D}\right)^{\frac{i}{i+2}} \tag{2}$$

$D = 3.65\times10^{-6} \mu m^{-2} s^{-1}$. The nucleation density is related to activation energy for diffusion (E_d) and can be expressed as:

$$N = exp\left(\frac{i}{i+2}\frac{E_d}{kT}\right) \tag{3}$$

For i = 1, following equation is used to calculate activation energy for diffusion (E_d):

$$N = exp\left(\frac{E_d}{3kT}\right). \tag{4}$$

We have also provided,

Table S1. Calculated Values for Diffusivity (*D*), and Activation Energy for Diffusion (E_d) for Four Different Substrates for *i*= 2

substrate	nucleation density (*N*) (μm^{-2})	diffusivity (*D*) (μm^{-2} s^{-1})	activation energy for diffusion, (E_d) (eV)
**MoS_2**	64.9 ± 2.46	2.37×10^{-4}	0.208
**graphene**	104.7 ± 3.21	9.12×10^{-5}	0.232
**SiO_2**	426.7 ± 2.83	5.49×10^{-6}	0.303

Table S2. Calculated Values for Diffusivity (*D*), and Activation Energy for Diffusion (E_d) for Three Different Thicknesses of MoS_2 for *i*= 2

substrate	nucleation density (*N*) (μm^{-2})	diffusivity (*D*) (μm^{-2} s^{-1})	activation energy for diffusion, (E_d) (eV)
**1L MoS_2**	64.9 ± 2.46	2.37×10^{-4}	0.208
**2L MoS_2**	61.0 ± 3.62	2.65×10^{-4}	0.205
**3L MoS_2**	52.7 ± 9.84	3.60×10^{-4}	0.198
**Bulk MoS_2**	12.5 ± 7.86	6.40×10^{-3}	0.126

Section 3: Layer dependent characteristics of In metal deposition on graphene

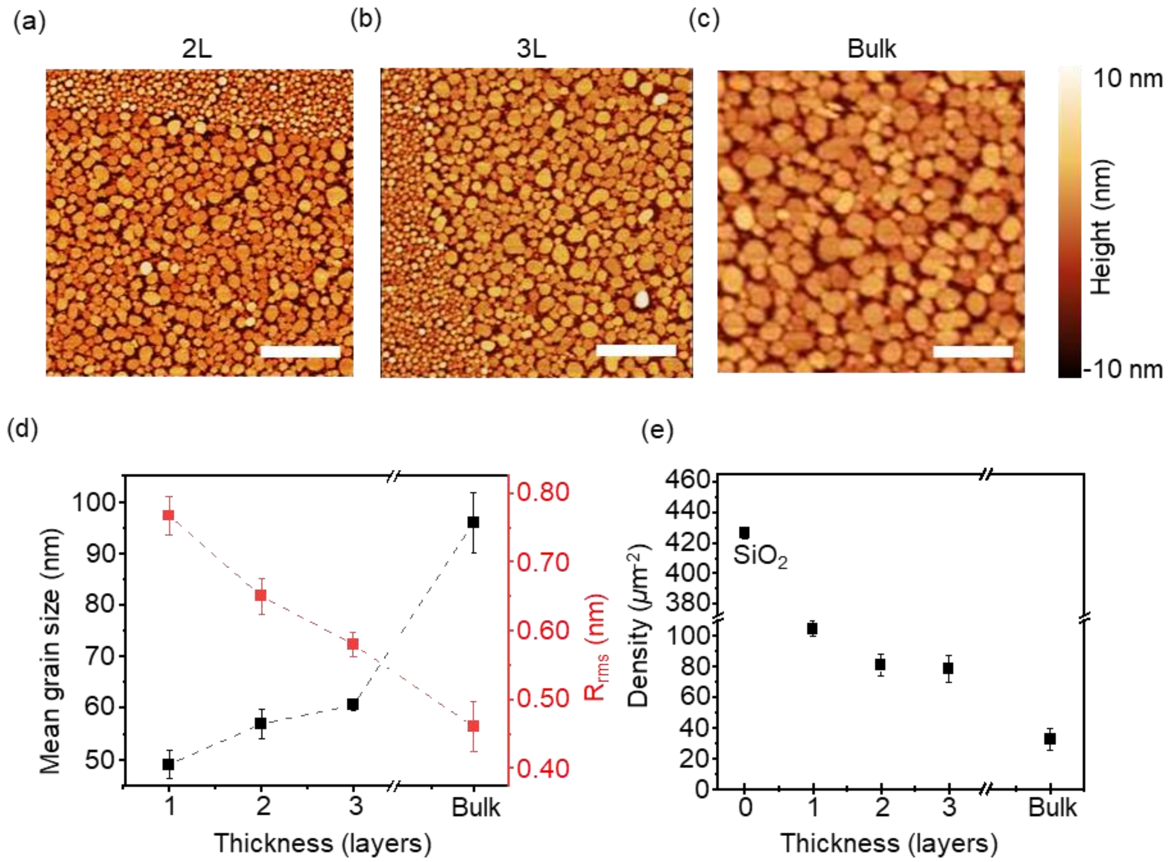

Figure S2. Morphology of In metal films deposited on different thicknesses of graphene. AFM (2×2 μm^2) images of 5 nm In deposited on (a) 2L, (b) 3L, and (c)Bulk graphene. Scale bar = 500 nm. (d) Mean grain size and R_{rms} (before metal deposition) of graphene flakes for different thicknesses of graphene. (e) In nucleation density on graphene for different thicknesses of graphene. Error bars in (d) and (e) represent the standard deviation across 5 AFM images (2×2 μm^2).

Table S3. Calculated Values for Diffusivity (*D*), Diffusion Length (L_D) and Activation Energy for Diffusion (E_d) for Three Different Thicknesses of Graphene (*i*= 1)

substrate	nucleation density (*N*) (μm^{-2})	diffusivity (*D*) (μm^{-2} s^{-1})	diffusion Length (L_D) (μm)	activation energy for diffusion, (E_d) (eV)
**IL**	104.7 ± 3.21	8.71×10^{-7}	0.098	0.349
**2L**	80.9 ± 6.78	1.89×10^{-6}	0.111	0.329
**3L**	78.8 ± 8.92	2.04 ×10^{-6}	0.113	0.328
**Bulk**	32.34 ± 7.43	2.95 ×10^{-5}	0.176	0.261

Table S4. Calculated Values for Diffusivity (*D*), and Activation energy for Diffusion (E_d) for Three Different Thicknesses of Graphene (*i*= 2)

substrate	nucleation density (*N*) (μm^{-2})	diffusivity (*D*) ($\mu m^{-2}\ s^{-1}$)	activation energy for diffusion, (E_d) (eV)
**IL graphene**	104.7 ± 3.21	9.12×10^{-5}	0.232
**2L graphene**	80.9 ± 6.78	1.53×10^{-4}	0.220
**3L graphene**	78.8 ± 8.92	1.61×10^{-4}	0.218
**Bulk graphene**	32.34 ± 7.43	9.56×10^{-4}	0.174

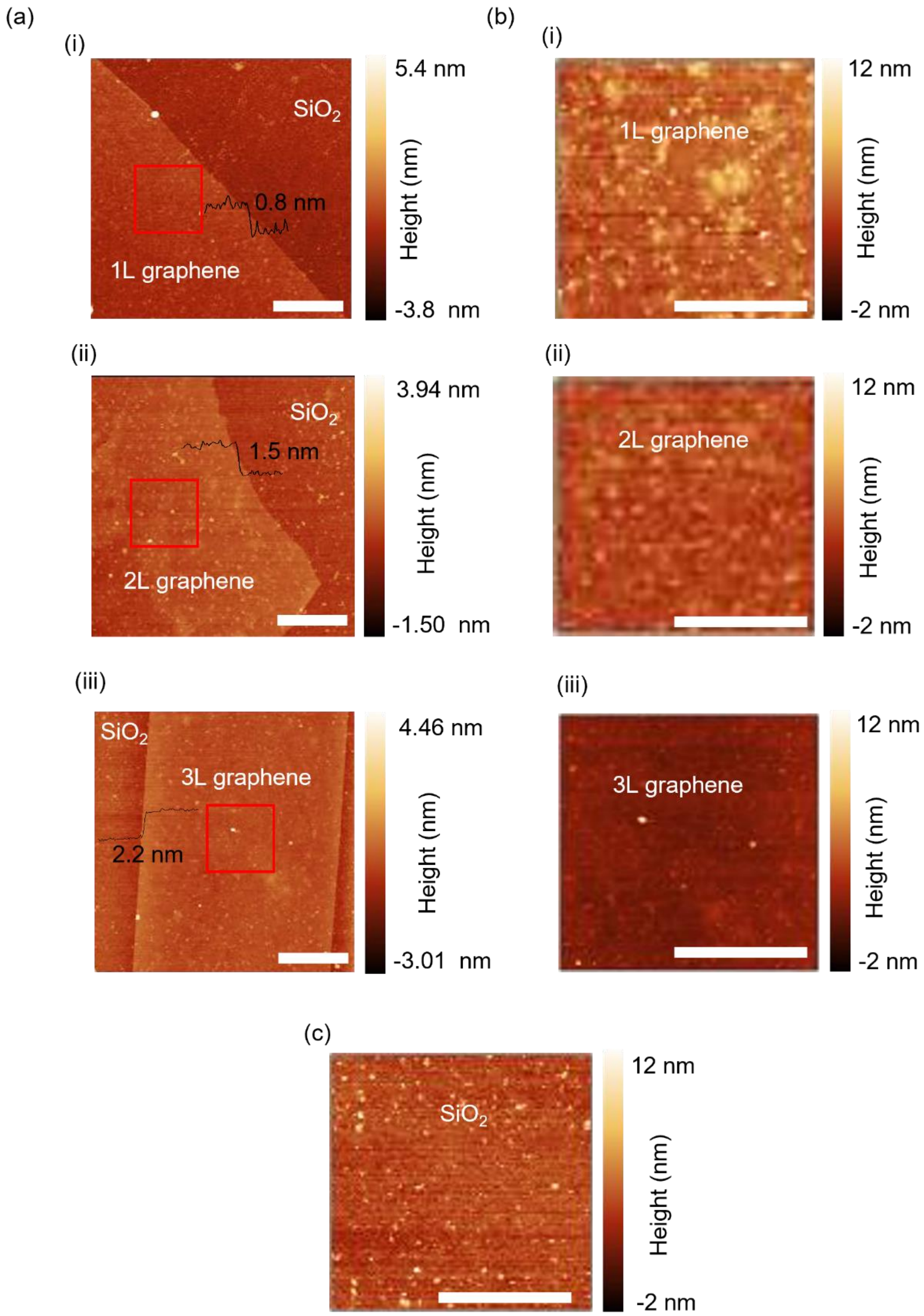

Figure S3. (a) AFM images (2×2 μm^2) of (i) 1L, (ii) 2L, (iii) 3L graphene flakes before metal deposition. Scale bar is 500 nm. AFM line profiles overlayed on the images indicate the thickness of the flake. (b) AFM images (500×500 nm^2) of bare graphene substrate regions marked inside red box from (a). (c) AFM image (500 ×500 nm^2) of bare SiO_2 substrate. Scale bar is 250 nm.

Section 4: Effect of thicker In metal deposition on grain morphology and Raman spectra

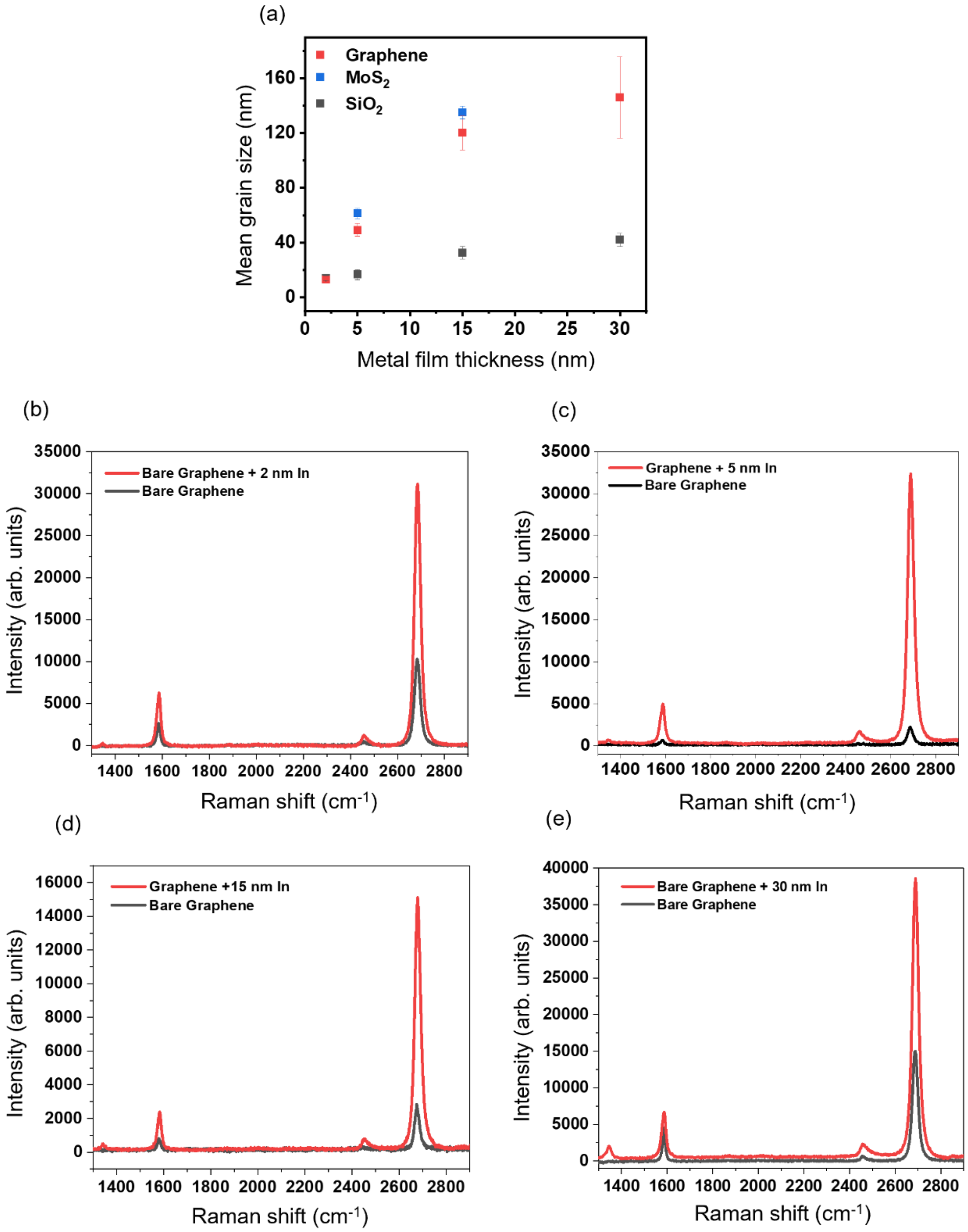

Figure S4. (a) Evolution of mean grain size for increasing thickness of In metal films on SiO_2, monolayer graphene, and monolayer MoS_2. Raman spectra of monolayer graphene collected after deposition of (b) 2 nm, (c) 5 nm, (d) 15 nm, (e) 30 nm thick In metal film deposition. Localized surface plasmon resonance effects are maximum for 5 nm thick In metal films.

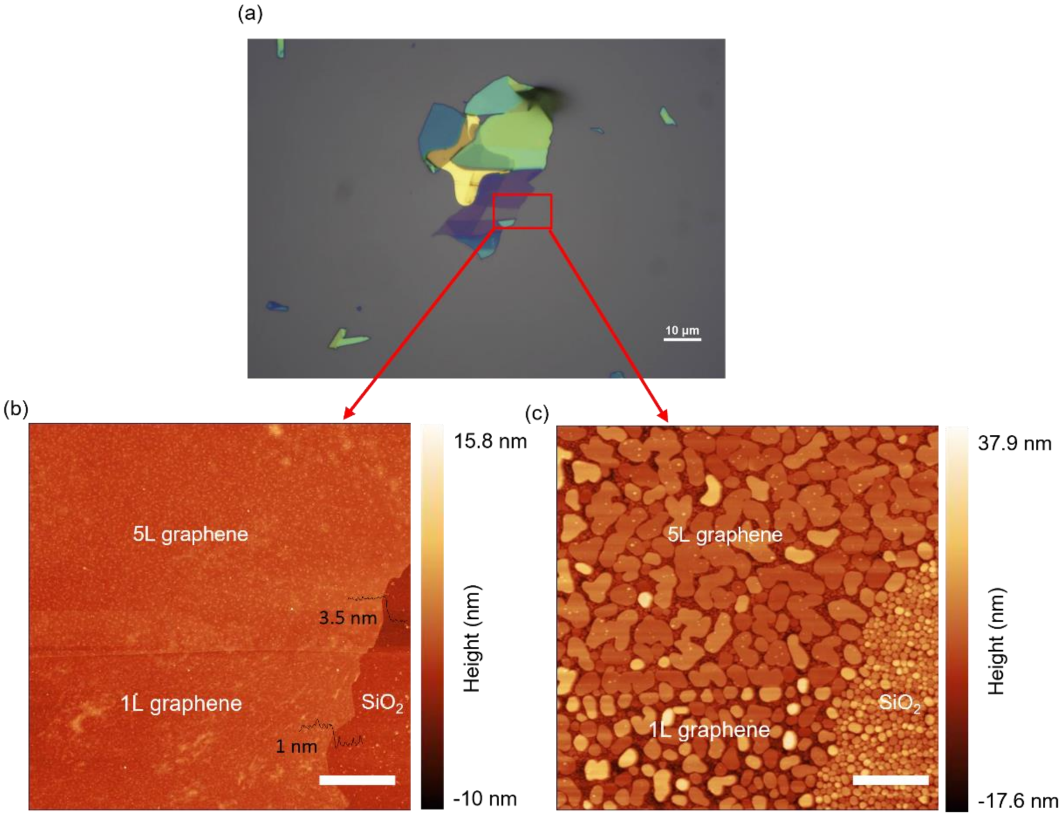

Figure S5. (a) Optical microscope image of a graphene flake with regions having different thicknesses. AFM image (5×5 μm^2) of region in (a) marked inside red box (b) before metal deposition and (c) after 15 nm thick In metal deposition. The image shows difference in grain size on different thicknesses of graphene, 1L graphene and 5L graphene, and SiO_2 substrate. Scale bar is 1 μm.

Section 5: Growth morphology of In and Au metal films on thermally annealed MoS_2 with additional defects

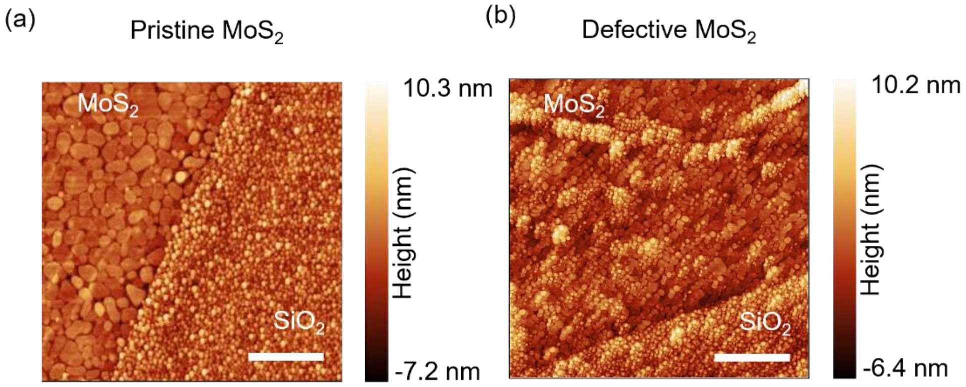

Figure S6. AFM (2×2 μm^2) image of 5 nm In deposited on (a) pristine and (b) defective monolayer MoS_2/SiO_2. Scale bar = 500 nm.

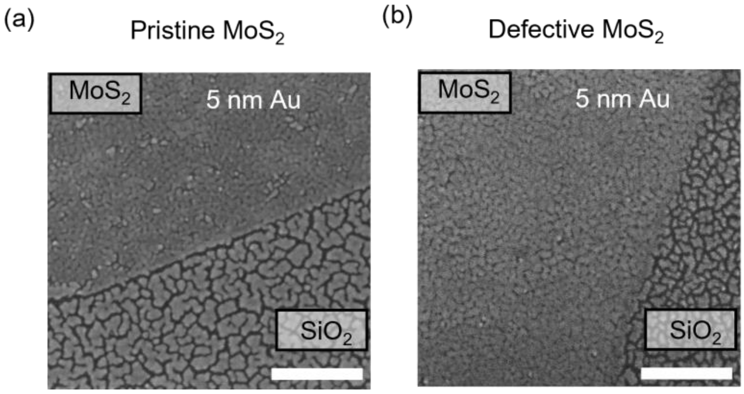

Figure S7. SEM image of surface morphology 5 nm Au film on (a) pristine and (b) defective MoS_2/SiO_2. Scale bar is 200 nm.

Section 6: Raman enhancement factor (EF) calculation

Raman enhancement factor for In coated 2D flakes was calculated using following equation,[2]

$$EF = \frac{I(In\ coated\ substrate)/A(hotspot)}{I(bare\ substrate)/A(excitation\ spot)} \tag{5}$$

I (*In coated substrate*) is the Raman peak intensity measured at 5 nm In coated MoS_2. We take *A(hotspot)* using the average radius (30 nm) of the In grains for 5 nm In coated MoS_2. *I(bare substrate)* is the MoS_2 Raman peak intensity measured at pristine flake without any metal deposition. The *A(excitation spot)* is the area of the laser excitation spot. We used 100× lens with 514 nm laser and used *A(excitation spot)* with radius of 450 nm.

Section 7: Differential reflectance of 1L MoS_2/5 nm Au

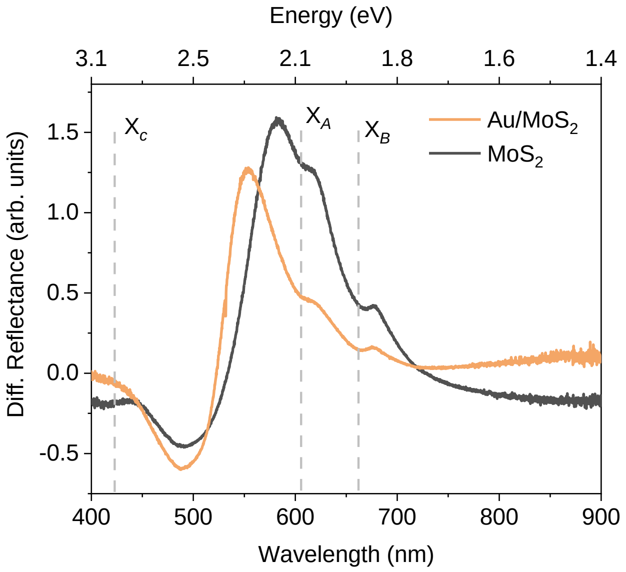

Figure S8. Differential reflectance of MoS_2/SiO_2 collected before and after 5 nm Au deposition.

REFERENCES

(1) Venables, J. A.; Spiller, T.; Hanbucken, M. Nucleation and Growth of Thin Films. *Rep. Prog. Phys*. **1984**, *47*, 399-459

(2) Xia, M.; Li, B.; Yin, K.; Capellini, G.; Niu, G.; Gong, Y.; Zhou, W.; Ajayan, P. M.; Xie, Y. H. Spectroscopic Signatures of AA' and AB Stacking of Chemical Vapor Deposited Bilayer MoS_2. *ACS Nano* **2015**, *9* (12), 12246–12254.
